# Real-World Patterns of Pharmacotherapeutic Management of Asthma Patients With Exacerbations in the Spanish National Health System

**DOI:** 10.3389/fphar.2020.01323

**Published:** 2020-08-21

**Authors:** Isabel Hurtado, Anibal García-Sempere, Salvador Peiró, Asier Bengoetxea, Jesús Luis Prieto, Gabriel Sanfélix-Gimeno

**Affiliations:** ^1^Health Services Research Unit, Fundación para el Fomento de la Investigación Sanitaria y Biomédica de la Comunidad Valenciana (FISABIO), Valencia, Spain; ^2^Red de Investigación en Servicios de Salud en Enfermedades Crónicas (REDISSEC), Valencia, Spain; ^3^Emergency Room Department, La Ribera University Hospital, Valencia, Spain; ^4^Medice Department, University of Valencia, Valencia, Spain

**Keywords:** asthma, exacerbation, pharmacotherapy, stepwise approach, real-world cohort, population-based

## Abstract

**Background:**

Little is known about the real1world characteristics of asthma patients with exacerbations or their pharmacotherapeutic management. We described the sociodemographic and clinical characteristics, and the patterns of short and long-term management of asthma attacks, in a population-wide cohort of exacerbators in the region of Valencia, Spain.

**Methods:**

We selected asthma patients with at least one exacerbation in 2015 and 2016, we classified them according to their patterns of exacerbations in the 4 years previous to the index exacerbation and their therapeutic step at baseline based on medication received in the previous year. We described the short and long-term pharmacological management of the index exacerbation.

**Results:**

18,714 patients experienced at least one exacerbation. The majority had no previous exacerbation (46.5%), or exacerbated in only one of the years (26.8%). 2.9% had attacks every single year, 25.7% of whom only received rescue medication at baseline. 29.5% of patients without previous exacerbation received maintenance therapy at baseline. Shortly following the index exacerbation, 2,461 patients (13.1%) did not receive any asthma prescription. Among those treated, 70.3% were prescribed a maintenance therapy, 62.4% received a rescue medication, and 30.5% received an oral corticoid. Throughout the year following the index exacerbation, most patients remained in their baseline therapeutic step.

**Conclusions:**

Most patients that exacerbate present very mild to mild forms of the disease or low levels of treatment and most exacerbations are managed in primary care. These insights may help to refine strategies for improving asthma control in the population.

## Introduction

Asthma is a chronic, long-term condition that causes intermittent inflammation and narrows the airways in the lungs, causing periods of wheezing, chest tightness, shortness of breath, and coughing. Asthma affects people of all ages and often starts during childhood, and symptoms, which may range from mild to severe, may present rarely or daily. When symptoms get worse, it is called an asthma attack or exacerbation (GEMA, 2019). Asthma is the most prevalent chronic respiratory disease and contributes notably to the global burden of disease of non-communicable diseases (To et al., 2012; GBD 2015 Chronic Respiratory Disease Collaborators, 2017; The Global Asthma Network, 2018). Recent estimations indicate that asthma is responsible for 1.1% of disability-adjusted life years (DALYs) worldwide (GBD 2016 DALYs and HALE Collaborators, 2017). From 1990 to 2015, global prevalence rose 12.6% and it is estimated that currently it affects 258.2 million people in the world, and roughly 10% of the European population (Bahadori et al., 2009). Data for Spain from the European Community Respiratory Health Survey (ECRHS)—commonly used as a reference in medical literature—show a prevalence of 5% for adults and 10% for children, though these data are now 20 years old and the situation may well have evolved (Grupo Español del Estudio Europeo en Asma, 1996; Urrutia et al., 2007).

The aim of the pharmacotherapeutic management of asthma is to maintain an adequate control of clinical symptoms, to control risk factors to prevent exacerbation and to minimize secondary effects from medication. Standard pharmacological treatments to control the disease include short and long acting ß2-agonists, inhaled and oral corticosteroids, and other therapies such as anticholinergic or biologic drugs that can be of interest for certain patients or situations. Despite the availability of effective treatment, there is compelling evidence of suboptimal control of asthma patients with asthma attacks in the real world, with important implications for the use of costly health care resources (Molina París et al., 2005; Peters et al., 2006; Martínez-Moragón et al., 2009). Inadequate asthma management is linked to increased risk of exacerbation, emergency room visits and hospital admissions, and higher mortality. Not surprisingly, patients with poorly controlled asthma suffer a poorer quality of life and incur in higher costs than well controlled patients (Doz et al., 2013).

Factors associated with exacerbation include asthma severity, gender, comorbidities, and a history of recent attacks (Blakey et al., 2017; Bloom et al., 2018b). Attacks can follow different patterns, appearing sporadically for some patients, grouped into series in response to seasonality changes or frequently recurring in the case of critical patients (Sears, 2008; Dougherty and Fahy, 2009; Kupczyk et al., 2014). This is relevant, as most studies on asthma attacks focus on patients at a higher risk, while an important burden of the social costs of asthma arise from a majority of patients with mild to moderate forms of the disease that are collectively responsible for considerable pharmaceutical costs and productivity losses (Aaron et al., 2008a; Aaron et al., 2008b; Royal College of Physicians, 2014; Aaron et al., 2017; Bloom et al., 2018a).

Available real-world evidence on the characteristics and management patterns of patients with asthma attacks in the Spanish National Health System is scarce. There is a lack of population-based studies and in most cases evidence comes from small prospective cohorts that may have observation and representativeness biases, thus limiting its extrapolation to the whole population of patients with asthma attacks. In the region of Valencia, an area of more than 5 million inhabitants, it is possible to carry out real-world data studies thanks to the Valencia Health System Integrated Database (VID), an integrated data framework that includes different clinical and administrative population-based databases that are linked at the individual patient level by means of a single identification number. The VID ecosystem allows for the follow-up of large population-wide cohorts for extended periods of time, thus offering an interesting opportunity to study the patterns of care of asthma patients with exacerbations. The aim of this study is to describe the sociodemographic and clinical characteristics of patients with asthma and exacerbations, and the patterns of short and long-term pharmacotherapeutic management of asthma attacks, based on a population-wide cohort of all patients with asthma exacerbations in the region of Valencia.

## Methods

### Design and Setting

This population-based cohort study was conducted in the Valencia Health System (VHS), a comprehensive structure of hospitals, primary care facilities, and other public resources managed by the Valencian regional government in Spain (more than 5 million inhabitants registered in 2010) providing free, universal healthcare services (apart from drug cost-sharing) to 97% of the region’s population. The VHS is organized territorially into 24 Health Departments (HDs). Each HD comprises one hospital and several primary care centers serving a population of between 150,000 and 250,000 people.

### Population

**Figure 1:**
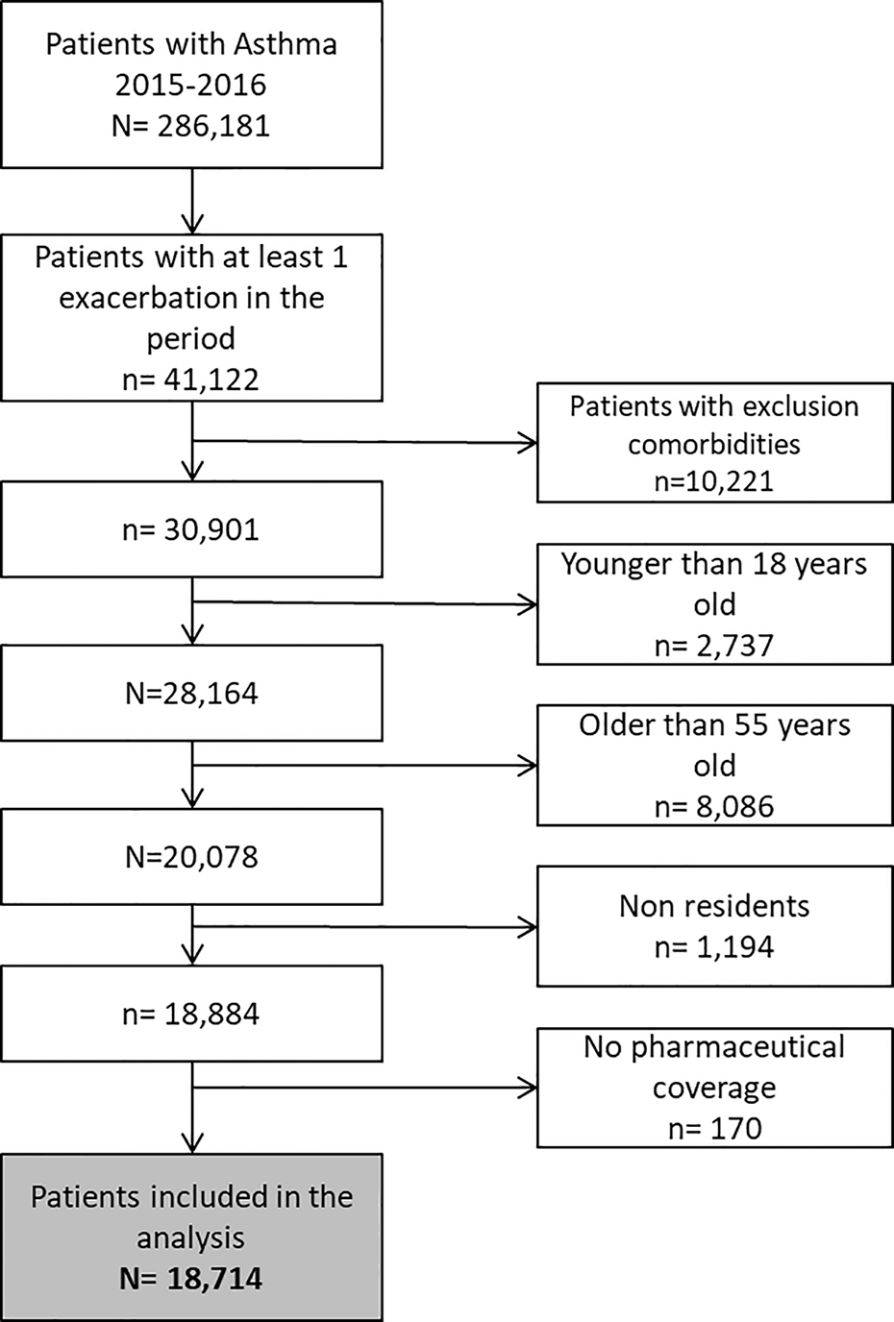
Flowchart.

We selected all the patients aged 18 to <56 years old with asthma (diagnosis code of International Classification of Diseases, Clinical Modification ICD9MC: 493.xx, ICD10MC: J45) in 2015 and 2016 that had an asthma exacerbation (index exacerbation) in the period and did not meet any of the exclusion criteria (see **Figure 1**. Flowchart and **Supplementary Material S1**).

### Data Sources

Data was obtained from the VID, a result of the linkage, by means of a single personal identification number, of a set of publicly-owned population-wide healthcare, clinical and administrative electronic databases in the region of Valencia, Spain, which has provided comprehensive information for the region’s approximately 5 million inhabitants since 2008. The VID includes sociodemographic and administrative information (sex, age, nationality, etc.) and healthcare information such as diagnoses, procedures, laboratory data, pharmaceutical prescriptions and dispensations, hospitalizations, mortality, healthcare utilization, and public health data (García-Sempere et al., 2020).

### Outcome Measures

The main outcome measures are the proportion of patients with a different history of exacerbation in each baseline therapeutic step, the proportion of patients in each therapeutic step in the year after the index asthma exacerbation (long-term management), and the patterns of short-term prescription in the month following the index exacerbation (short-term management).

### Covariates

Variables potentially related to asthma and the risk of exacerbation in the study population over the study period were considered. These included socio-demographic characteristics, comorbidities, and healthcare resource utilization in the preceding 12 months (see **Supplementary Material S2** for coding detail on covariates).

### Definition of Exacerbation

We employed seven definitions for exacerbation, as these can be managed in different levels of care. Moreover, we included four different definitions for exacerbations attended to in hospital emergency rooms due to variable coding practices in this setting. To account for exacerbations with multiple contacts with the system, we considered that exacerbations recorded within 15 days of each other were part of the same episode. We used the highest level of care required by an episode to classify exacerbations, ranked from a higher to a lower level of care: hospital admission, hospital emergency room, primary care emergency room, specialized care, and primary care (see **Supplementary Material Table S3**).

### Definition of Patterns of Past Exacerbation

We established a look-back period of 4 years from the index exacerbation to describe the individual patterns of past exacerbations, and we used the following definitions: a “sporadic pattern” was assigned to patients with at least one exacerbation in only one of the 4 years; a “recurrent pattern” was defined as having at least one exacerbation in more than 1 year but not in all years; a “frequent pattern” phenotype was defined as at least one exacerbation in each year. Patients with no exacerbation at all in the 4 year period were classified as “no exacerbation.”

**Figure 2:**
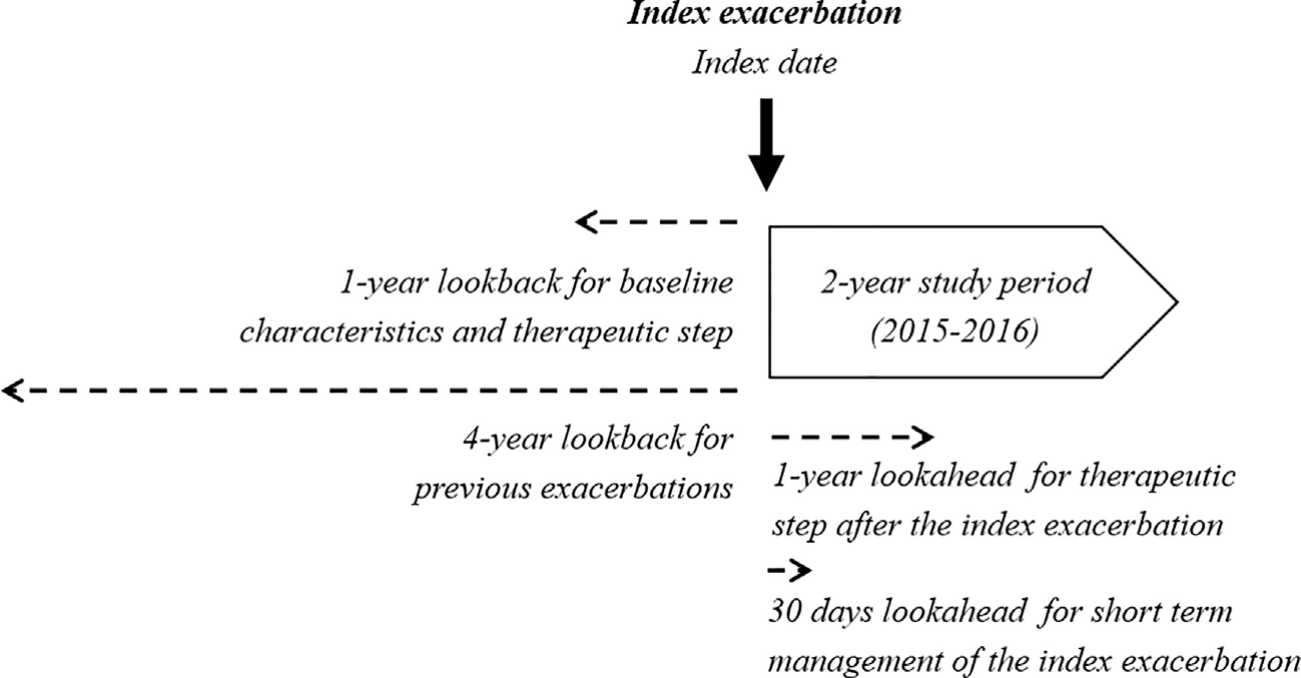
Time frames employed for assessment.

**Figure 2** summarizes the different time frames employed to perform all the analyses.

### Classification of Asthma Medications

We classified every single prescription into four different categories: maintenance therapy, rescue medication, attack medication, and chronic obstructive pulmonary disease (COPD) medication (see **Supplementary Material Tables S4** and **S5**). To classify the prescriptions containing inhaled glucocorticoids (IGC) by strength (low, medium, or high), we first calculated the ICG dosing of each prescription with the help of the dosage schedule specified by the physician for each treatment. We then classified each ICG-containing prescription according to the GEMA beclomethasone equivalence table (see **Supplementary Material Table S6**).

### Definition of Therapeutic Steps

To measure asthma severity and to classify patients by therapeutic step at baseline and in the year after the index exacerbation, we based our analysis on the 2019 Spanish Guidelines for Asthma (GEMA 4.4 (GEMA, 2019)) stepwise approach (incorporating medication class and dose), an evidence-based method of measuring asthma severity, similar to the British Thoracic Society stepped approach that has been employed in the same way in other settings (Bloom et al., 2018a; Bloom et al., 2018b). We used all prescriptions in the year before the index exacerbation date, excluding the 30 days immediately before and after the index date, and we estimated the periods of the year covered by the different therapeutic steps. We then allocated each patient the predominant step in terms of coverage of a longer number of days. We had to adapt the steps in GEMA to the reality of the management patterns found in real-word clinical practice.

We were able to classify 99.5% of patients in GEMA steps at baseline and post-exacerbation based on medication. We considered steps 5 and 6 in the GEMA guidelines as a single step 5 and 6, mainly due to the overlapping definition of these steps in GEMA, making it difficult to differentiate between step 5 and 6 patients in clinical practice. We created an additional category called “maintenance therapy <180 days,” for a relatively small group of patients with an unexpected prescription pattern based on maintenance medication (but covering fewer than 180 days a year) with not a single prescription for rescue medication throughout the year. We did not consider this uncommon treatment pattern in the description of findings. **Table S7** in **Supplementary Material** describes in detail the procedure followed.

### Analysis

First, we described patient characteristics. Second, we described patterns of exacerbation in the 4 years prior to the exacerbation date. Third, we described the severity of the asthma in the year before the index exacerbation by therapeutic step (baseline therapeutic step). Fourth, we described the therapeutic step in the year after the index exacerbation. Fifth, we calculated the percentage of patients with different patterns of past exacerbation in different baseline therapeutic steps. Sixth, we described the relation between the therapeutic steps before and after the index exacerbation.

### Ethics

The study protocol was approved by the regional Ethics Committee for Clinical Research of the Hospital Clinic Universitari (25-03-2019). Informed patient consent was not required because datasets were extracted with anonymized identifiers according to Spanish laws on privacy (Act 3/2018) and patients’ rights (Act 41/2002).

## Results

### Patient Characteristics

A total of 18,714 patients with asthma experienced at least one exacerbation meeting the definition in **Supplementary Material Table S3** in the period 2015 to 2016. Mean age was 36.8 years old and 61% were female patients. The most frequent comorbidities were rhinitis (49.4%), anxiety (38.8%), sinusitis (28.7%), atopy (20.9%), depression (13.3%), and hypertension (9.2%). Also, 21.4% of patients reported smoking. We had BMI data for 10,104 patients, of whom 60.9% had a BMI>25 kg/m^2^. 22.5% of patients attended an ER more than twice in the year before the index exacerbation, and 11.6% had at least one hospital admission in the same period. 9.2% visited a pneumologist and 13.9% visited an allergology specialist in the years preceding the index attack. With regard to medication, 61.8% of patients received at least one short-acting beta agonists (SABA) prescription and 27% received at least one oral corticoid prescription in the year before the exacerbation date. By baseline therapeutic step as defined in **Table 2**, 37.6% of patients received no asthma medication in the year before the index exacerbation, 25.3% were in step 1 with no maintenance therapy, 12,2% were in step 4 and 5.4% were in combined step 5 and 6. The main patient characteristics are described in **Table 1**.

**Table 1:** Patients characteristics at baseline, number, and percentage.

		N= 18,714	
**Socio-demographics (n, %)**			
Gender	Women	11,413	60.99
	Men	7,301	39.01
Age (population between 18 and <56 years old)	=<25	3,236	17.00
	26-35	4,78	25.54
	36-45	5,946	31.77
	>=46	4,752	25.39
BMI	<18.5	380	2.03
	18.5–25	3,450	18.44
	25–30	2,896	15.48
	30–40	2,758	14.74
	>=40.0	620	3.33
	Unknown	8,610	46.01
Country	Spain	15,207	81.26
	Europe	850	4.54
	Others	2,657	14.20
Health care utilization (12 months previous to the index date)			
Primary care visits	0–4	6,344	33.90
	5–12	6,967	37.23
	>=13	5,403	28.87
Specialist visits	0–4	15,180	81.12
	5–12	2,851	15.23
	>=13	683	3.65
Nursing visits	0–4	17,181	91.81
	5–12	1,122	6.00
	>=13	411	2.20
ER visits	0	9,653	51.6
	1	4,851	25.9
	>=2	4,210	22.50
Hospital visits	>1	16,541	88.39
Neumology visits	>1	1,726	9.22
Allergology visits	>1	2,603	13.91
Medication (12 months previous to the index date, 3 months previous for NSAID)			
NSAID	>1	5,304	28.34
SABA	>1	11,559	61.77
Oral corticoids	>1	5,053	27.00
Comorbidities (12 months previous to the index date)			
Myocardial infarction		90	0.48
Hypertension		1,728	9.23
Dementia		43	0.23
Diabetes		551	2.94
Depression		2,497	13.34
Anxiety		7,267	38.83
Cancer		661	3.53
Liver disease		985	5.26
Kidney disease		77	0.41
Sinusitis		5,378	28.74
Rhinitis		9,251	49.43
Conjunctivitis		524	2.80
Reflux		1,166	6.23
Sleep apnea		481	2.57
Food allergy		387	2.07
Drugs allergy		442	2.36
Atopy		3,905	20.87
Dermatitis		806	4.31
Lifestyle (12 months previous to the index date)			
Sedentary lifestyle		8	0.04
Smoking		4,007	24.41
Alcohol		491	2.62
Baseline therapeutic step (based on medication in the 12 months before the index date			
No treatment		7,046	37,63
Maintenance <180 days		875	4,67
Step 1		4,738	25,30
Step 2		1,165	6,22
Step 3		1,592	8,50
Step 4		2,280	12,18
Step 5–6		1,008	5,38
Not classified		20	0,11

**Table 2:** Level of care required for index asthma attacks (N: number of subjects, %: percentage).

	N	%
Hospital admission	369	1.97
Hospital emergency room	4,219	22.54
Primary care emergency room	11,639	62.19
Specialized care	138	0.74
Primary care	2,349	12.55
**Total**	**18.714**	**100**

### Index Exacerbations per Definition

From 18,714 index exacerbations, almost 2% required admission to hospital, 22.5% were attended to in hospital ERs, and the majority (62.2%) were managed in primary care emergency room facilities. 12.5% of exacerbations were attended to in regular primary care visits and only 0.7% were managed by specialists in regular ambulatory visits (**Table 2**).

### Patterns of Exacerbation and Baseline Therapeutic Step

The majority of patients had no exacerbations in the 4 years previous to the index exacerbation (46.5%), or exacerbated in only 1 year (26.8%), and 2.9% were frequent exacerbators that suffered from attacks every single year. In the year before the index exacerbation, frequent exacerbators were predominantly treated in step 4 (29.9%), while 20.6% were in the higher treatment step 5 and 6 and 25.7% received only rescue medication. For recurrent and sporadic patterns, almost a third of the patients were in step 1 at baseline, receiving only rescue medications (30.4 and 29% respectively), and an additional 26.7% of sporadic exacerbators did not receive any medication in the year previous to the index exacerbation (14.9% for recurrent exacerbators). Patients without attacks in the 4 years previous to the index exacerbation were managed mostly with no treatment (46.5%) or only rescue medication (23.1%), while 29.5% received maintenance therapy in the year before the index attack (see **Table 3**).

**Table 3:** Previous patterns of exacerbation pattern according to therapeutic step at baseline, n, and (%).

	No treatment	Step 1	Step 2	Step 3	Step 4	Step 5 and 6	Maintenance <180 days	Not classified	Total
No exacerbation	5,638 (46.55)	2,796 (23.08)	651 (5.37)	875 (7.22)	1,106 (9.13)	434 (3.58)	600 (4.95)	12 (0.10)	12,112
Sporadic pattern	1,040 (26.76)	1,128 (29.03)	296 (7.62)	398 (10.24)	582 (14.98)	254 (6.54)	184 (4.73)	4 (0.10)	3,886
Recurrent pattern	359 (14.87)	734 (30.39)	199 (8.24)	278 (11.51)	499 (20.66)	256 (10.60)	87 (3.60)	3 (0.12)	2,415
Frequent pattern	9 (2.89)	80 (25.72)	19 (6.11)	41 (13.18)	93 (29.90)	64 (20.58)	4 (1.29)	1 (0.32)	311
Total	7,046	4,738	1,165	1,592	2,280	1,008	875	20	18,724

Patterns of exacerbation are defined with a lookback period of 4 years before the index exacerbation. Baseline therapeutic step is defined by the medications received in the 12 months preceding the index exacerbation.

### Baseline Therapeutic Step and Post-Exacerbation Therapeutic Step (Long-Term Exacerbation Management)

Throughout the year following the index exacerbation, the majority of patients remained in their baseline therapeutic step (ranging from 60.4% of baseline step 5 and 6 patients to 37.5% of baseline step 2 patients). For patients in step 1 at baseline (only rescue medication), 32.4% were predominantly treated in steps 2 to 6 in the year following the index attack. For patients in step 4 at baseline, the treatment step rose to steps 5 and 6 in 7% of these patients after the attack, while 20.6% of patients in step 3 at baseline were treated in steps 4, 5, and 6 in the following year. For patients in step 5 and 6 at baseline, 39.9% were predominantly treated in lower steps over the year after the index exacerbation (see **Table 4**).

**Table 4:** Therapeutic step in the year after the index exacerbation according to baseline therapeutic step, n, and (%).

	No treatment	Step 1	Step 2	Step 3	Step 4	Step 5&6	Maintenance <180 days	Not classified	Total
No treatment	3,854 (54.70)	1,373 (19.49)	289 (4.10)	411 (5.83)	552 (7.83)	162 (2.30)	397 (5.63)	8 (0.11)	7,046
Step 1	1,086 (22.92)	1,880 (39.68)	332 (7.01)	425 (8.97)	572 (12.07)	209 (4.41)	209 (4.41)	25 (0.53)	4,738
Step 2	163 (13.99)	198 (17.00)	437 (37.51)	113 (9.70)	155 (13.30)	61 (5.24)	34 (2.92)	4 (0.34)	1,165
Step 3	165 (10.36)	184 (11.56)	92 (5.78)	785 (49.31)	253 (15.89)	75 (4.71)	34 (2.14)	4 (0.25)	1,592
Step 4	206 (9.04)	287 (12.59)	111 (4.87)	211 (9.25)	1,246 (54.65)	161 (7.06)	41 (1.80)	17 (0.75)	2,280
Step 5 and 6	54 (5.36)	117 (11.61)	40 (3.97)	40 (3.97)	121 (12.00)	609 (60.42)	19 (1.88)	8 (0.79)	1,008
Maintenance <180 days	318 (36.34)	174 (19.89)	41 (4.69)	62 (7.09)	85 (9.71)	34 (3.89)	154 (17.60)	7 (0.80)	875
Not classified	2 (10.00)	8 (40.00)	0	2 (10.00)	4 (20.00)	0	1 (5.00)	3 (15.00)	20
Total	5,848	4,221	1,342	2,049	2,988	1,311	889	76	18,724

Baseline steps in rows, post-exacerbation steps in columns.

Baseline therapeutic step is defined by the medications received in the 12 months preceding the index exacerbation. Therapeutic step in the year after the index exacerbation is defined by the medication received in the 12 months following the index exacerbation.

### Short-Term Exacerbation Management

In the 30 days immediately following the index exacerbation, 2,461 patients (13.1%) did not receive any prescription for asthma medication. From the 16,263 patients that received at least one prescription for asthma, 70.3% were prescribed a maintenance therapy, 62.4% received a rescue medication, and 30.5% received a prescription for oral corticoids. Among those patients receiving maintenance therapy, 79% received an IGC/long-acting β2-adrenergic agonist (LABA) combination, 22% received leukotriene receptor antagonists (LTRA), and 17.6% were prescribed an IGC monotherapy prescription.

## Discussion

In this population-based cohort of 18,724 patients with asthma that had at least one asthma exacerbation in the period 2015–2016 in the region of Valencia, we found that almost half of the patients had no attacks in the 4 years preceding the index exacerbation, and roughly half of the patients had no medication or only rescue medication prescribed in the year before the index attack. This confirms that the vast majority of patients that exacerbate present very mild to mild forms of the disease or low levels of treatment, in line with available evidence worldwide (Lai et al., 2003; Gibson et al., 2013; Bloom et al., 2018a; Bloom et al., 2018b). Accordingly, strategies for improving the management of asthma patients should undoubtedly take into account these patients, and not only severe asthma cases? The distribution of exacerbations in our setting by levels of care reinforces this recommendation for action. We showed that, in addition to the widely studied attacks that require hospitalization or ER care, two thirds of exacerbations are managed by primary care emergency services. By using a multidimensional definition of exacerbations, we may have captured mild to moderate exacerbations that do not require hospital level care, but are extremely relevant in terms of burden of disease, healthcare costs, and quality of life losses, and which are not usually captured in studies on asthma attacks. We will quantify this impact in further studies based on this same cohort.

In general terms, the relation between history of exacerbation and baseline therapeutic step was reasonable, with patients with a worse history being proportionally treated in higher steps. Even if our analysis does not allow for appropriateness assessment, it may provide some warning signs. In this sense, the fact that roughly 20% of the patients with a 4 year history of no exacerbation were treated at baseline in steps 3 to 6 may be a call for a review of the management of these patients with a maintenance therapy. This potential overtreatment, however, did not prevent them from exacerbation. Perhaps of greater concern may be the fact that more than a quarter of frequent exacerbators—patients with exacerbation in each of the 4 years before the index attack—were using no maintenance medication or received no treatment at all in the year previous to the attack, suggesting the presence of a potential problem of underuse in high-risk patients. To our knowledge, this is the first study that provides population-based, real-world evidence on the characteristics of asthma patients with exacerbations and the patterns of phamacotherapeutic care delivered to these patients in the Spanish National Health Service.

With regard to the relationship between the baseline step and post-attack treatment step, we observed that the majority of patients remained in the same therapeutic step in the year following the index attack. This could be signaling a phenomenon of clinical inertia, but further analyses are needed to confirm this suspicion. Also, when looking at the short-term management of exacerbations, we found that a concerning proportion of patients did not receive any medication for asthma in the 30 days following the exacerbation, again raising the question of whether some underuse or clinical inertia may be occurring in the presence of asthma exacerbations. We will analyze the relation between history of exacerbation, therapeutic step and risk of future exacerbation in an upcoming study based on the same cohort to shed some light on appropriateness and clinical inertia. Finally, almost two-thirds of patients exacerbating were female, in line with international evidence and sex differences in asthma prevalence. Accordingly, programs for improving the quality of asthma care should incorporate the gender perspective.

### Limitations

Our study is subject to some limitations. First, we excluded COPD patients to minimize misclassification between COPD and asthma exacerbations. In this way our analysis does not include a relevant group of patients with concomitant COPD and asthma, but this was necessary to adequately capture asthma attacks. Second, we lack information on out-of-pocket medications. A proportion of rescue medication, especially salbutamol, may be used out-of-pocket, which may result in misclassification in steps based on medication. Also, we may be missing exacerbations that are self-managed by patients with out-of-pocket medication, resulting in a potential underestimation of the number of mild to moderate exacerbations and the use of rescue medication. However, this proportion may well be small and thus may be affecting our classification only marginally. Third, use of formoterol combinations was classified as maintenance therapy, but these medications can also be used as reliever therapy. Accordingly, they are not included in our definition of rescue medication. Fundamental changes in clinical practice could occur in the near future, as clinical guidelines may be shifting toward recommending maintenance-and-reliever therapeutic schemes in mild to moderate patients at the expense of the traditional “SABA on demand and no maintenance” approach, and render this operational definition obsolete (Reddel et al., 2019). Fourth, despite including many relevant individual variables in our analysis, we may have omitted important variables such as the/an eosinophil count or Asthma Control Test scores as these data are not routinely recorded in linkable clinical databases, though their absence does not affect the relevance of our results. Fifth, information biases due to absent registration or differing data-recording practices in the electronic databases might exist, although this is an inherent problem of any study using data from routine clinical practice. Sixth, we based our analysis on prescription information but excluded refills. In this way, we may be overestimating exposure as real-world adherence to chronic medication is generally suboptimal, though our aim was not to calculate effective exposure to treatments but rather identify the treatment step as defined by prescribing doctors. Seventh, we did not assess the association of patterns of exacerbation and management with clinical outcomes, typically the occurrence of recurrent exacerbations. This analysis is due to be performed shortly after the present study.

## Conclusion

We found that the majority of patients presented mild to moderate forms of the disease in terms of history of exacerbation and medication used, and also that the majority of exacerbations were not attended to in the hospital setting. These findings may help refine strategies for improving asthma control in the population. Further research will be undertaken shortly to analyze the relation between exacerbations and health services utilization, productivity losses and mortality, and between history of exacerbation and therapeutic step of treatment with the risk of recurrent exacerbations.

## Data Availability Statement

The datasets presented in this article are not readily available because legal restrictions on sharing the data set apply as regulated by the Valencia regional government by means of legal resolution by the Valencia Health Agency [2009/13312] which forbids the cession of data to third parties (accessible at: http://www.san.gva.es/documents/152919/157920/resolucionsolicituddatos.pdf). Upon request, authors can allow access to the databases in order to verify the accuracy of the analysis or the reproducibility of the study. Requests to access the datasets should be directed to Management Office of the Data Commission in the Valencia Health Agency (email: solicitud_datos@gva.es; telephone numbers: +34 961-928207; +34 961-928198).

## Ethics Statement

The studies involving human participants were reviewed and approved by Regional Ethics Committee for Clinical Research of the Hospital Clinic Universitari. Written informed consent for participation was not required for this study in accordance with the national legislation and the institutional requirements.

## Author Contributions

IH, AG-S, SP, and GS-G were responsible for the study concept, design and data acquisition. IH carried out the data preparation and the statistical analysis and AG-S drafted the manuscript. IH, AG-S, SP, GS-G, JP, and AB participated in the analysis and interpretation of data, critical revision of the manuscript for important intellectual content, approved the final version submitted for publication and agree to be accountable for all aspects of the work in ensuring that questions related to the accuracy or integrity of any part of the work are appropriately investigated and resolved.

## Funding

This study was funded by the 2018 Collaboration agreement between FISABIO, a research body depending from the Health Department of the Valencia Government, and GlaxoSmithKline S.A., to conduct independent research on “Patterns of clinical management of asthmatic exacerbations in real clinical practice in the National Health System”. The funding sources had no access to the study data nor participate in the design or conduct of the study, data analysis, decisions regarding the dissemination of findings, writing of the manuscript, or decisions about its publication. The views presented here are those of the authors and not necessarily those of the FISABIO Foundation, the Valencia Ministry of Health, or the study sponsors.

## Conflict of Interest

The authors declare that the research was conducted in the absence of any commercial or financial relationships that could be construed as a potential conflict of interest.
