# Supplementary material for: Real-World Patterns of Pharmacotherapeutic Management of Asthma Patients With Exacerbations in the Spanish National Health System
Source: Front Pharmacol. 2020 Aug 21;11:1323. doi: 10.3389/fphar.2020.01323 (PMC7472631; doi:10.3389/fphar.2020.01323)
Supplement: Supplementary file 1 [file DataSheet_1.docx]

**Real-world Patterns of Pharmacotherapeutic Management of Asthma Patients with Exacerbations in the Spanish National Health System**

**Supplementary Material**

| **Table S1. Exclusion criteria** |
| --- |
| 1. Patients younger than 18 years old |
| 2. Patients older than 55 years old in the moment of the index exacerbation |
| 3. Patients with emphysema, codes CIE9MC: 492.8; CIE10ES: J43.9 |
| 4. Cystic fibrosis, codesCIE9ES:277.00; CIE10ES:E84.9 |
| 5. Congestive heart failure, codes CIE9MC 428.xx, 398.91, 402.01, 402.11, 402.91, 404.01, 404.11, 404.03, 404.13, 404.91, 404.93; CIE10ES: I50 |
| 6. Lung cancer, codes CIE9MC: 162.x; CIE10ES: C34.9- C78.0- D02.2- D14.3- D38.1 D49.1 |
| 7. COPD, codes CIE9MC: 496, 491.21, 493.20, 494; CIE10ES: J44, J47. |
| 8. Patients without pharmaceutical/health coverage by the VHA, mainly some government employees whose prescriptions are reimbursed by civil service insurers and thus not included in the pharmacy databases of the VHA |
| 9. Patients not registered in the municipal census (non-residents or temporary residents), or those who left the region or were disenrolled from VHA coverage for other causes due to  limitations on follow-up |

| **Table S2. Definition of covariates** | | |
| --- | --- | --- |
| *Covariate* | *ICD9-CM* - *International Classification of Diseases, Ninth Revision, Clinical Modification* | *ICD10-ES -* *International Classification of Diseases, Tenth Revision, Spanish adaptation* |
| Rhinitis | 472.0x, 477.xx | J31.0x, J30.xx |
| Sinusitis | 477.9x, 473.xx | J32.xx |
| Reflux | 530.81 | K21.0x, K21.9x |
| Apnea | 786.03, 780.51, 780.53, 780.57 | G47.3x, R06.8x |
| Aspergillosis | 518.6x | B44.81 |
| Food allergy | 693.1x, V15.01, V15.02, V15.03, V15.04, V15.05 | T78.1x |
| Conjunctivitis | 372.14 | H10.44, H10.45 |
| Atopic dermatitis | 691.8x | L20.xx |
| Drug allergy | 995.27, V14 | Z88.xx |
| Diabetes | 249.xx, 250.xx | E10.xx, E11.xx, E13.xx |
| Hypertension | 401.xx, 402.xx, 403.xx, 404.xx, 405.xx | I10.xx, I11.xx, I12.xx, I13.xx, I15.xx |
| Alcohol use | 291.xx, 303.xx, 305.0x, 980.0x, 760.71 | F10.xx, T51.xx, G62.1x |
| Dementia | 290.xx, 294.xx, 330.xx, 331.xx | F03.xx |
| Depression | 296.2x, 296.3x, 298.0x, 300.4x, 301.12, 311.xx | F34.1x, F32.xx, F33.xx |
| Malignancy | 140.xx-190.xx, 200.xx, 201.xx-208.xx, 23.xx | C0.xx- C9.xx, D0.xx, D3.xx, D4.xx |
| Kidney disease | 585.xx, 584.xx, 586.xx, 403.xx, V45.1 | I12.xx, I13.xx, N17.xx-N19.xx, Z91.15 |
| Liver disease | 570.xx, 572.xx, 571.5x, 571.6x, 571.8x, 571.9x, 573.0x, 573.4x, 573.8x, 573.9x, 782.4x, 789.1x, 789.5x, 790.4x, 790.5x, 794.8x, V42.7 | K70.xx, K71.xx, K72.xx, K73.xx, K74.xx, K76.xx |
| Coronary Heart Disease | 410.xx – 414.xx | I20.xx-I25.xx |
| Atopy | 995.3x, V15.09 | T78.40XA |
| Smoking | 989.84, 305.1x | O99.33, Z72.0x, T65.2x, F17.2x, Z87.891 |
| Sedentary lifestyle | V69.0 | Z72.3x |
| Anxiety | 293.84, 309.24, 300.0x, 308.0x | F41.xx |

| **Table S3. Definitions of exacerbation employed in this study.** |
| --- |
| 1. Hospitalization coded with a diagnose of asthma. |
| 2. Emergency Room (ER) attendance coded with a diagnose of asthma. |
| 3. ER attendance coded with a diagnose of acute bronchospasm, in patients that have an active code of asthma previous to the ER visit or activated in the 30 days following the visit. |
| 4. ER attendance without coding, in patients:  - without any asthma medication in the year previous to the ER visit.  - that receive an asthma medication within 15 days from the visit:: SABA, ipratropium, IGC, IGC/LABA, IGC/SABA, LTRA, tiotropium, teofilline, oral corticoid (metemos las de epoc aquí?).  - that have an active code of asthma previous to the ER visit or activated in the 30 days following the visit. |
| 5. ER attendance without coding in patients with a previous active code of asthma or activated in the 30 days following the ER visit, that initiate treatment with oral corticoid or SABA and ipratropium within 15 days of the ER visit. |
| 6. Primary care emergency room visit coded with a diagnose of asthma, or coded with a diagnose of acute bronchospasm in patients with a previous active code of asthma or activated within 30 days of the visit. |
| 7. Specialist or primary care visit coded with a diagnose of asthma exacerbation (ICD9CM: 493.x2, ICD10ES not necessary as only used for inhospital coding) |
| 7bis. Specialist or primary care visit coded with a diagnose of acute bronchospasm in patients that have an active code of asthma previous to the visit or activated in the 30 days following the visit. |

*SABA: short-acting B agonists, IGC: inhaled glucocorticoids, LABA: long-acting B agonists, LTRA: leukotriene receptor antagonists.*

| **Table S4. Medications included(ATC codes and INN) under different groups** | |
| --- | --- |
| *Groups* | *Medication included* |
| SABA | Salbutamol, terbutaline, bambuterol |
| LABA | ATC R03AC (excluding salbutamol, terbutaline, bambuterol and clembuterol) |
| GCI | ATC R03BA |
| GCI/LABA | ATCR03AK |
| Oral corticoids | ATC H02,oral formulations only |
| LTRA | Montelukast, zafirlukast |
| Rescue, other tan SABA | Ipratropium, ipratropium/salbutamol, beclomethasone/salbutamol |
| COPD | Aclidinium, aclidinium/formoterol, glycopyrronium, glycopyrronium/indacaterol, umeclidinium, vilanterol, umeclidinium/vilanterol, formoterol/beclomethasone/glycopyrronium, olodaterol/tiotropium |
| Cromoglicic acid | Cromoglicic acid, disodium cromoglycate, nedocromil |
| Biologics | Mepolizumab, omalizumab, reslizumab |
| Other medication included, not grouped | Tiotropium, Theophylline, Terbutaline/Guaiphenesin |

*SABA: short-acting B agonists, IGC: inhaled glucocorticoids, LABA: long-acting B agonists, LTRA: leukotriene receptor antagonists, COPD: chronic obstructive pulmonary disease, ATC: Anatomical Therapeutic Chemical (ATC) Classification System, INN: international nonproprietary name.*

| **Table S5. Classification of drugs and drug groups into medication categories.** | | | |
| --- | --- | --- | --- |
| *Medication category* | *Drugs included* | *Rationale* | *Aims* |
| Maintenance therapy | IGC, IGC/LABA, LTRA, Tiotropium, Theophylline, Cromoglicic acid, Terbutaline/Guaiphenesin, Biologics | Drugs indicated as asthma maintenance indication and recommended by GEMA | Identification of attacks; Allocation of patients to therapeutic steps; description of short term management of attacks. |
| Rescue medication | SABA, Ipratropium, GCI/SABA, SABA/Ipratropium | Drugs indicated as rescue and attack management medication in asthma and recommended by GEMA | Identification of attacks; Allocation of patients to therapeutic steps; description of short term management of attacks. |
| Attack medication | Oral Corticoids | Drugs indicated solely to treat exacerbations or in Step 6 and recommended by GEMA | Identification of attacks; Description of short term management of attacks. Allocation of Step 6 patients into Step 5&6 category. |
| COPD medication | LABA, LABA/LAMA, other drugs with COPD-only indication | In clinical practice, COPD medications are being prescribed for some asthma patients | We considered these medications as asthma maintenance therapy where rescue medication was also used. Patients with COPD medication with no rescue medication were allocated to the “Maintenance <180d” step in Table S4 |

*SABA: short-acting B agonists, IGC: inhaled glucocorticoids, LABA: long-acting B agonists, LTRA: leukotriene receptor antagonists, LAMA: long-acting muscarinic antagonist, COPD: congestive obstructive pulmonary disease, GEMA: Spanish guideline for asthma management.*

| **Table S6. Equipotent doses of ICG according to GEMA Guidelines** | | | |
| --- | --- | --- | --- |
|  | Low dose (ug/day) | Medium dose (ug/day) | High dose (ug/day) |
| Beclomethasone dipropionate | 200-500 | 501-1.000 | 1.001-2.000 |
| Beclomethasone extrafine | 100-200 | 201-400 | > 400 |
| Budesonide | 200-400 | 401-800 | 801-1.600 |
| Ciclesonide | 80-160 | 161-320 | 321-1.280 |
| Fluticasone furoate | - | 92 | 184 |
| Fluticasone propionate | 100-250 | 251-500 | 501-1.000 |
| Mometasone furoate | 100-200 | 201-400 | 401-800 |

*From Guia Española para el Manejo del Asma, 2019.*

| **Table S7. Classification of patients in therapeutic steps according to medication** | | | |
| --- | --- | --- | --- |
| Steps in our study | GEMA Guideline medication based steps | Allocation criteria in our study | Additional information |
| Step 1 | - No maintenance therapy - SABA on demand | - Rescue medication: at least one prescription of SABA | - Patients with at least on prescription of SABA in the year and no other medication were allocated to Step 1. |
| Step 2 | - Maintenance: Low dose IGC or LTRA - SABA on demand | - Low dose ICG or LTRA> 180 days - Rescue medication | - When no step covered at least 180 days, we allocated the step covering a longer number of days. |
| Step 3 | - Maintenance: low dose ICG+LABA or medium dose ICG or low dose ICG+LTRA | - More than 180 days covered by any of the possible step 3 maintenance options - Rescue medication |  |
| Step 4 | - Medium dose ICG+LABA or LTRA | - More than 180 days covered by any of the options covered by step 4 - Rescue medication |  |
| Step 5&6 | - Two separated steps with overlap - High dose IGC+LABA - + Tiotropium or Teofilline or LTRA - + Biologics - + Oral Corticoids | - More than 180 days covered by any of the options covered by steps 5&6 - Rescue medication | - We merged Steps 5 and 6 in one single step due to the overlapping definition in GEMA and the low number of patients. |
| Maintenance therapy < 180d | - Not in GEMA | - No rescue medication - Maintenance therapy <180d + COPD-only therapy | - Peculiar pattern found in clinical practice, difficult to classify. |
| No treatment | - Not in GEMA | - No asthma prescriptions |  |
| Not classified | - Not in GEMA | - Patterns unclassifiable | - Erratic prescription patterns, difficult to classify |

*SABA: short-acting B agonists, IGC: inhaled glucocorticoids, LABA: long-acting B agonists, LTRA: leukotriene receptor antagonists, COPD: chronic obstructive pulmonary disease, GEMA: Spanish guideline for asthma management.*
